# Do hip-abduction braces work?—A biomechanical evaluation of a commercially available hip brace

**DOI:** 10.1007/s00402-021-03989-8

**Published:** 2021-06-13

**Authors:** Roman Michalik, Katrin Essing, Ben Rohof, Matthias Gatz, Filippo Migliorini, Marcel Betsch

**Affiliations:** 1 Department of Trauma and Reconstructive Surgery, University Hospital RWTH Aachen, Pauwelsstraße 30, 52074 Aachen, Germany; 2 Department of Orthopaedic Surgery, University Hospital RWTH Aachen, Aachen, Germany; 3 Department of Orthopaedics and Trauma Surgery, University Medical Center Mannheim of the University Heidelberg, Mannheim, Germany

**Keywords:** Hip brace, Inertial sensors, Hip dislocation, Total hip arthroplasty, Arthrodesis cushion

## Abstract

**Introduction:**

Dislocations of the hip joint are a common and clinically relevant complication following total hip arthroplasty (THA). Hip-abduction braces are currently used following operative or non-operative treatment of THA dislocations to prevent re-dislocations. However, the clinical and biomechanical effectiveness of such braces is still controversial.

**Material and methods:**

A total of 30 volunteers were measured during standing and during sitting up and down from a chair task wearing a hip brace set at 70°, 90° or no hip flexion limitation. Range of motion of the hip joint was measured in all directions by an inertial sensor system. Further it has been evaluated if the range of motion would be reduced by the additional use of an arthrodesis cushion.

**Results:**

The use of a hip brace set up with flexion limitation did reduce hip ROM in all directions significantly compared to unhinged brace (*p* < 0.001–0.035). Performing the “sit down and stand-up task” the brace set up at 70° flexion limitation did reduce maximum hip flexion significantly (*p* = 0.008). However, in most cases the measured hip flexion angles were greater than the settings of the hip brace should have allowed. The additional use of a cushion can further limit hip motion while sitting up and down from a chair.

**Conclusion:**

This study has demonstrated that hip-abduction braces reduce hip range of motion. However, we also found that to achieve a flexion limitation of the hip to 90°, the hip brace should be set at a 70° hip flexion limitation.

## Introduction

Dislocations of the hip joint after total hip arthroplasty (THA) remain one of the most common postoperative complications. In the literature dislocation rates after primary THA are reported between 1.7% and 2.2% [1, 2], whereas dislocations after revision surgery appear in 5.1–5.7% of the cases [2, 3]. Recent research even suggests that these numbers are substantially understated [4]. Risk of re-dislocation of THA is reported in the literature to be as high as 20–40% [5–7].

Risk factors for a THA dislocation can be divided into surgery-dependent and patient-dependent factors. Among the most common surgery-dependent risk factors for THA dislocation are the surgical approach [8], soft tissue tensioning, component positioning [9], implant impingement, femoral head size and surgeon experience are among the most common causes [10].

Several patient-dependent risk factors for THA dislocations have been identified including age over 70 years, muscle weakness, soft tissue laxity, non-compliance, cognitive or neuromuscular disorders and prior surgeries as a spinal fusion [11, 12].

First-time THA dislocations can often be managed non-operatively by closed reduction of the THA and the post-interventional use of a hip-abduction brace. Most physicians use a hip brace for 6–12 weeks post-reduction [10, 13]. The small number of studies evaluating the use of hip abduction braces does, however, report controversial recommendations. A study by Ishii et al. showed a benefit of hip braces in preventing dislocation after primary THA. The authors indicate, that the brace helps patients recognizing provocative positions for dislocation and follow functional restrictions [10]. In contrast report, DeWal et al. no difference in the re-dislocation rate of patients wearing or not wearing a brace after THA dislocation [3].

Besides the small number of studies evaluating the clinical effectiveness of hip braces in preventing THA dislocation, there is even less information about the effectiveness of hip-abduction braces in reducing hip range of motion. Therefore, the purpose of this study was to examine and biomechanically investigate a commonly used hip-abduction brace in healthy volunteers in terms of its hip range of motion limiting function. Hypothesis of this study is that the use of a hip-abduction brace will significantly decrease hip range of motion.

## Material and methods

### Human participants

A sample size estimation (90% power, level of significance 5%, and an effect size of 0.6) revealed that 30 participants were to be included in this study for sufficient statistical power (Software G*Power, Version 3.1, HHU Düsseldorf, Germany). Healthy individuals between the age of 18 and 75 years were enrolled. Exclusion criteria were known musculoskeletal deformities or past injuries of the spine, pelvis or lower extremities, a BMI above 35 kg/m^2^ (as it may affect brace fitting and measurement accuracy) chronic diseases such as i.e. rheumatoid arthritis or diabetes and the inability to safely stand or walk. The study protocol was approved by the local ethics committee (EK 091/17) and all volunteers gave their oral and written consent to participate in this study.

### Materials

**Fig. 1:**
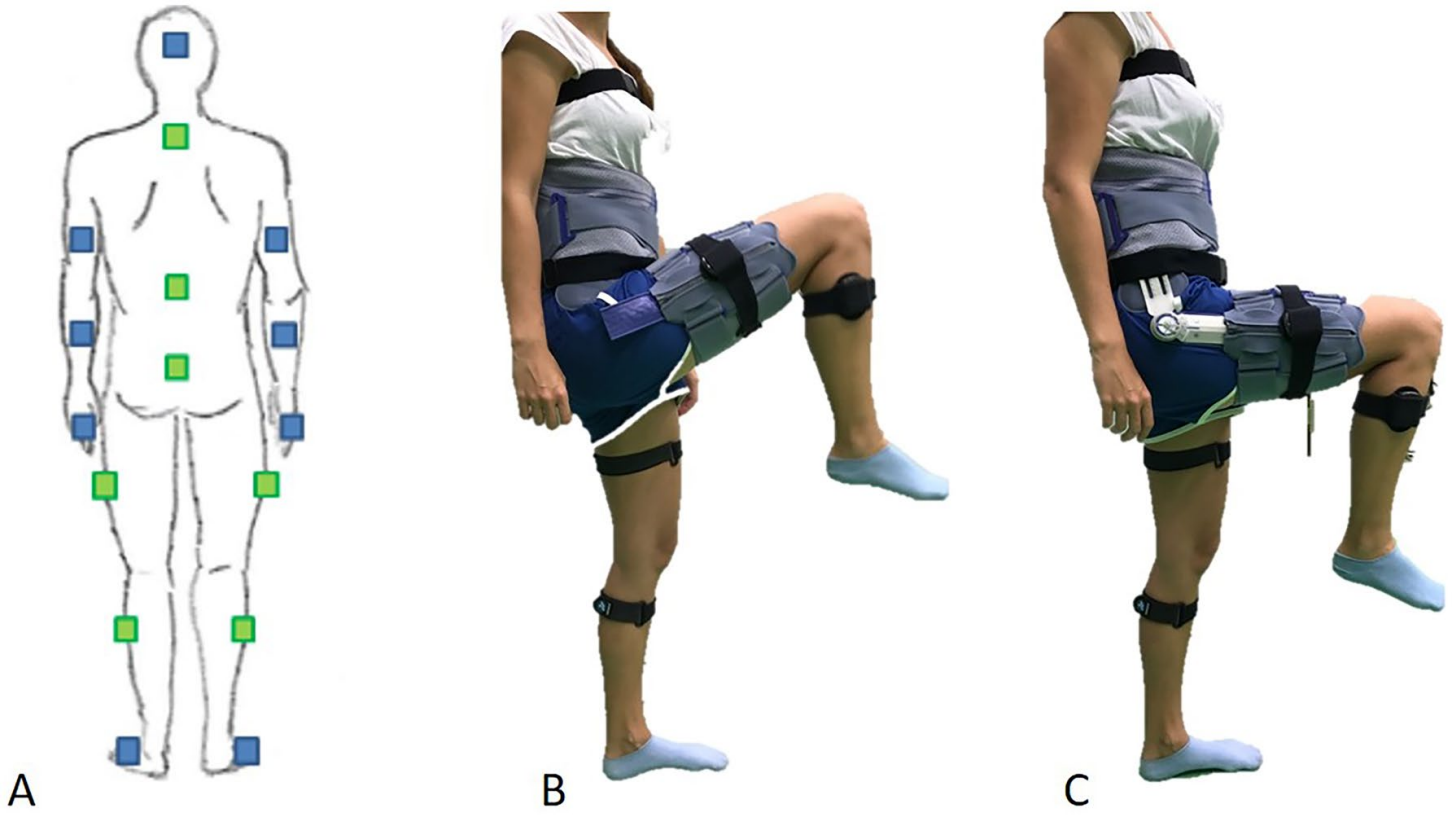
**A** The hip range of motion was measured with an inertial sensor system. The sensors were placed according to the green marked spots. **B** The hip abduction brace (SoftTec^®^ Coxa, Bauerfeind^®^, Germany) was placed with the hinge at the level of the hip joint center. The unhinged setting allows unrestricted flexion of the hip. **C** The hip brace allows an adjustment of flexion limitation. The construction of the brace further limits rotation, ab-and adduction and extension of the hip

A commercially available hip-abduction brace (SofTec^®^ Coxa; Bauerfeind^®^, Zeulenroda-Triebes, Germany) was used in our measurements, which has a waist-and thigh-band that can be adjusted with hook-and-loop straps (Fig. 1). The hinge of the hip brace consists of an adjustable metal joint with both hip flexion and abduction stops. In all subjects, the hip brace was placed so that the hinge was positioned at the level of the hip joint center according to the manufacturer’s instructions. The pelvic circumference was measured in all subjects, to choose the correct size of the hip brace and to fit the hip brace according to the subject’s anatomy. Mean pelvic circumference was 91.1 cm (SD 6.2). Straps were fixed with tension on the pelvic shells so that the brace could not displace. Straps around the thigh were adapted to thigh circumference and adjusted for a firm fit.

The hip motion of the volunteers was measured with an inertial sensor system (Myomotion; Noraxon, Scottsdale, AZ, USA). This system uses sensors, which are attached to the human body with special straps on two adjacent body segments to calculate the joint range of motion (ROM) between these segments by tracking the sensor’s 3D-angular orientation. The sensors include 3D accelerometer, gyroscope and magnetometer to measure the rotational angles of each sensor. They transmit motion of the human body to a specific receiver to compute angular changes of the measured joints with a frequency of 100 Hz and an accuracy of ± 2% [14]. This system has been tested for validity and reliability in several studies [14–16]. For the study, seven sensors were placed in a standardized fashion on the femur and lower thigh of both legs, as well as on the pelvis, upper and lower thorax (Fig. 1).

### Measurement protocol

The following brace settings were tested in this study because they represent clinically relevant restrictions after THA: (1) Unhinged brace setting (control setting) (2) hip flexion limited to 90° and (3) hip flexion limited to 70° (Fig. 1).

**Fig. 2:**
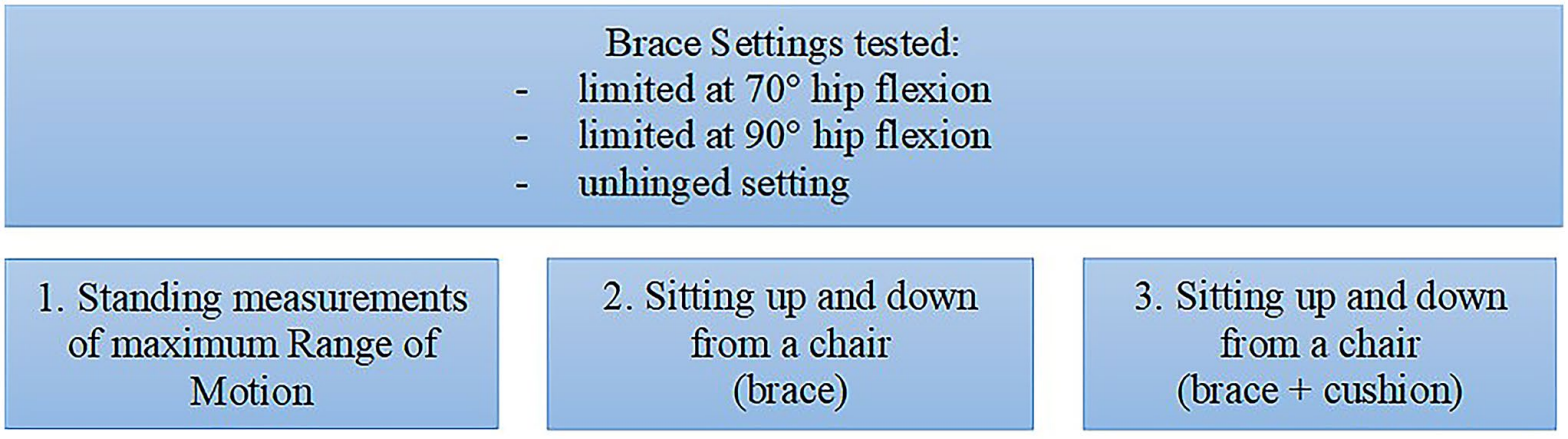
Flowchart of measurements taken in the study: Subjects were measured wearing a hip brace in the unhinged, 70°and 90° flexion limitation setting while performing movement of the hip in the standing position while sitting up and down from a chair, and finally while performing the same task with additional use of an arthrodesis cushion

To evaluate the effectiveness of the hip brace, the following movements during upright standing of the volunteers have been tested: maximum hip extension/flexion, hip abduction/adduction and hip external/internal rotation. All volunteers were instructed to maximally move their leg in the respective directions (Fig. 2).

**Fig. 3:**
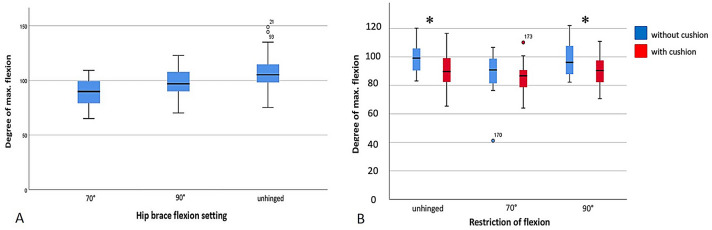
**A** Boxplot graphic showing the maximum flexion (in degree) measured in three settings (unhinged, 90° and 70° flexion limitation) of the hip brace in standing position. Analysis revealed significant differences in the maximum hip flexion between all three settings (*p* < 0.001). **B** Boxplot graphics display the maximum hip flexion measured while wearing a hip-brace only (red boxes) and during the additional use of an arthrodesis cushion (blue boxes). Significant differences (*) were measured with an unhinged brace (*p* < 0.001) and with the 90° setting of the hip brace (*p* < 0.001)

Then, we tested the three brace settings while sitting up and down from a chair with a defined height of 45 cm. Finally, all volunteers were evaluated for hip ROM while sitting down and standing up from a chair with a seat raiser (cushion) under the measured hip with the respective settings. Figure 3 gives an overview over the performed measurements.

### Statistical analysis

Statistical analysis was performed using Matlab (Version 2018a, MathWorks^®^ Inc., Natick, MA, USA) and SPSS software (IBM SPSS Statistics, Version 24, Chicago, IL, USA). All data were checked for Gaussian distribution using the Kolmogorov–Smirnov test. We used a one-way ANOVA for repeated measurements (RMANOVA) with a modified post-hoc Bonferroni test. The level of significance was set at *p* < 0.05.

## Results

The average age of the subjects was 28.4 years (SD 9.4) with an average height and weight of 174.6 cm (SD 10.1) and 69.6 kg (SD 12.6), respectively. The demographic data of our subjects is summarized in Table 1. Results of the standing ROM measurements showed that the hip brace significantly limits hip ROM. Maximum flexion of the hip joint while standing without any restriction of hip flexion (unhinged brace) was 107.4° (SD ± 17.8°). Maximum hip flexion was significantly reduced to 99.2° (SD ± 12.6°) (*p* = 0.001) with the brace set to 90° and, respectively, reduced to 89.4° (SD 11.1°) (*p* < 0.0001) with the brace set to 70° flexion (Fig. 3A). The difference of maximum hip flexion between the two restricted settings (90° vs. 70°) was also significant (*p* < 0.0001).

**Table 1:** Demographic data of all subjects

	Total (*n* = 30)	Male (*n* = 14)	Female (*n* = 16)
Age (years)	28.6 ± 9.4	29.25 ± 9.65	27.43 ± 9.01
Height (cm)	174 ± 10.1	181.49 ± 8.18	166.43 ± 4.11
Weight (kg)	69.6 ± 12.6	77.94 ± 10.77	60 ± 6.07
BMI (kg/m^2^)	22.7 ± 2.9	23.67 ± 2.65	21.74 ± 2.86
Pelvic circumference (cm)	91.1 ± 6.2	94.63 ± 5.68	87.14 ± 3.94

Both hip brace settings (70° and 90°) led to a significant reduction of the maximum hip abduction and adduction when compared to the unhinged brace setting (*p* < 0.001). However, we did not find a significant difference in maximum hip abduction between the 70° and 90° brace settings (*p* = 1.00) (Table 2). The maximum external and internal rotation of the hip during standing was also significantly reduced between the two settings (70° and 90°) and the unhinged brace setting (*p* < 0.001). For the maximum internal hip rotation there was also a difference between the 70° and 90° setting (*p* = 0.026). However, we did not find a significant reduction in maximum external hip rotation between the 70° and 90° setting (*p* = 0.255) (Table 2).

**Table 2:** Measured mean maximum range of motion and corresponding *p* values of the hip wearing the hip abduction brace in unhinged or flexion-limited settings

Flexion	Unhinged	90° limit	70° limit	Extension	Unhinged	90° limit	70° limit
Mean/SD	107.4 ± 17.8	99.2 ± 12.6°	89.4 ± 11.1	Mean/SD	21.0 ± 8.8	17.7 ± 5.8	18.3 ± 7.4
Unhinged	–	*p* = 0.001	*p* < 0.001	Unhinged	–	*p* = 0.035	*p* = 0.022
90° limit		–	*p* < 0.001	90° limit		–	*p* = 1

Abduction	Unhinged	90° limit	70° limit	Adduction	Unhinged	90° limit	70° limit
Mean/SD	40.2 ± 9.9	21 ± 9.5	21.1 ± 8.6	Mean/SD	19.4 ± 8	9.5 ± 6.8	9.1 ± 6.3
Unhinged	–	*p* < 0.001	*p* < 0.001	Unhinged	–	*p* < 0.001	*p* < 0.001
90° limit		–	*p* = 1	90° limit		–	*p* = 1

Ext. Rot	Unhinged	90° limit	70° limit	Int. Rot	Unhinged	90° limit	70° limit
Mean/SD	41.8 ± 8.9	32.5 ± 8.4	34.2 ± 8.2	Mean/SD	32.6 ± 7.5	28.1 ± 7.4	25.2 ± 7
Unhinged	–	*p* < 0.001	*p* < 0.001	Unhinged	–	*p* = 0.001	*p* < 0.001
90° limit		–	*p* = 0.255	90° limit		–	*p* = 0.026

Next, we evaluated the influence of the hip brace on hip motion during sitting up and down from a chair. We did find a significant reduction in maximum hip flexion between the 70° and unhinged (*p* = 0.008) as well as between the 70° and 90° (*p* = 0.002) hip brace setting. There was no difference in maximum hip flexion between the 90° and unhinged brace setting (*p* = 1.00) (Table 3). For all other hip motions (abduction, adduction, external and internal rotation), we did not find significant differences between different brace settings (*p* = 0.887–1.0).

**Table 3:** Measured mean maximum range of motion performing a “sitting up and down from a chair task” while wearing a hip abduction brace (with unhinged or flexion limiting setting).

Brace setting	Hip flexion No cushion	Hip flexion With cushion	*p* value
Unhinged	99 ± 10	89.7 ± 11.6	*p* < 0.001
90° limitation	97.8 ± 10.4	90.2 ± 9.2	*p* < 0.001
70° limitation	88.9 ± 12.7	85.7 ± 9.7	*p* = 0.231

Brace setting	Hip abduction No cushion	Hip abduction With cushion	
Unhinged	8.3 ± 7.8	11 ± 8.8	*p* = 0.002
90° limitation	10.1 ± 8.3	11.2 ± 8.4	*p* = 0.205
70° limitation	10.1 ± 10.5	10.6 ± 9.4	*p* = 0.567

Brace setting	Hip adduction No cushion	Hip adduction With cushion	
Unhinged	5.1 ± 4.8	5 ± 5.35	*p* = 0.837
90° limitation	4.6 ± 6.5	4.2 ± 6	*p* = 0.406
70° limitation	5.6 ± 6.47	4.7 ± 6.4	*p* = 0.289

Brace setting	Hip ext. rotation No cushion	Hip ext. rotation With cushion	
Unhinged	9.3 ± 8.17	9.7 ± 6.6	*p* = 0.746
90° limitation	10.5 ± 8	12 ± 7	*p* = 0.180
70° limitation	11.33 ± 6.9	12.08 ± 5.8	*p* = 0.544

Brace setting	Hip int. rotation No cushion	Hip int. rotation With cushion	
Unhinged	7.5 ± 7.6	7.2 ± 6.4	*p* = 0.771
90° limitation	5.8 ± 6.5	4.3 ± 5.9	*p* = 0.036
70° limitation	5.8 ± 7.7	4.1 ± 4.4	*p* = 0.133

We evaluated whether or not the additional use of an arthrodesis cushion results in a significant limitation of the hip range of motion

Finally, we have evaluated the effects of a cushion placed on the chair during sitting up and down. By using the cushion in addition to the hip brace, we did find significant differences (cushion versus no-cushion) of the maximum hip flexion for the unhinged (*p* < 0.001) and 90° hip (*p* < 0.001) brace setting (Fig. 3B). Rotation was reduced by the use of the arthrodesis cushion, which was significant only for internal rotation with a brace set up at 90° flexion limitation (*p* = 0.036). Abduction and adduction were mostly not reduced by the additional arthrodesis cushion. Wearing the unhinged brace, hip abduction was even significantly higher with the use of a cushion (*p* = 0.002).

## Discussion

In this study, we were able to show that hip abduction braces can effectively limit the ROM of the hip joint. Furthermore, we were able to show, that an arthrodesis cushion can also limit hip flexion when used.

A dislocation of the THA is a major postoperative complication after THA with a significant impact on the patient’s rehabilitation [1, 2]. While the dislocation rate has been reported in the recent literature to be about 2% [1, 2], re-dislocation rate after closed reduction is reported to be as high as 20–40% [5–7]. One way to reduce the risk of re-dislocation and increase joint stability after non-operative and operative treatment of a THA dislocation is the temporary immobilization or the use of motion limiting hip braces [13, 17–19]. A further benefit of hip braces is that they can create awareness in patients to limit their ROM and to limit their motions that can lead to a dislocation of the joint. However, so far the use of a hip brace is controversial because its effectiveness has only been evaluated in clinical studies with a low number of patients and no biomechanical studies do yet exist [3, 10, 17, 19].

In this present study, we have examined the effects of a commercially available hip-abduction brace on the hip ROM during standing and while sitting up and down from a chair using an inertial sensor system. Healthy volunteers were enclosed instead of patients with a THA, due to the possible risk of sustaining an actual hip dislocation in patients associated with the measurements of ROM and movements performed. Our results showed that a brace setting of 70° or 90° hip flexion has led to a significant reduction in hip motion compared to the unhinged brace setting. However, our findings also demonstrated that the actual maximum hip flexion was greater than the set flexion limitation of the brace. These findings are of great relevance because to achieve a clinical limitation to 90° of hip flexion the hip brace should be set at a 70° flexion limitation instead of a 90° flexion limitation. An explanation for this can be that metal hinge of the brace cannot fully withstand forces of the hip and leg during motion. Furthermore, it is possible that the hip brace cannot be adjusted perfectly to the participant’s anatomy. However, we took great care in assuring the correct position of the hinge over the center of hip rotation, as well as making sure that the straps were positioned and tightened in accordance with the manufacturer’s instructions. It could also be that even when fitted perfectly there is some residual motion between the brace and the hip/leg of the participants. In previous studies, it was also confirmed that orthopedic braces, which are used for other joints might also not be able to fully resist the musculoskeletal forces so that the targeted restriction or support cannot always be fully achieved [20, 21].

The results of our study show that setting a flexion limitation of 70° or 90° also significantly limits the abduction, adduction, internal and external motion of the hip joint of up to 50%.

While superior and anterior THA dislocations are associated with adduction of the extended hip joint, the posterior THA dislocation is usually provoked by deep flexion of the hip joint [22]. Studies have shown that dorsal/posterior dislocations are the most common THA dislocations [17]. A finite element analysis on hip dislocation provoking maneuvers by Nadzadi et al. revealed that the “sit to stand” maneuver is considered to be the most provocative for a dislocation [23]. Based on these findings, we chose to study the effects of a hip brace on hip ROM during a “sitting up and down” from a chair task.

Here we found that the brace setting of 70° led to a significant reduction of hip flexion in comparison to the unhinged setting while standing up from a chair. To further limit the risk associated with deep hip flexion, the use of an elevated toilet seat or raised chair are often standard postoperative precautions after total hip replacement and especially after THA dislocation [24, 25]. In our study, we have evaluated the effectiveness of a so-called hip arthrodesis cushion in addition to the use of a hip brace. Our results showed that the combination of a cushion with a hinged hip brace set at 90° further limits hip flexion during sitting up and down from a chair, which supports the use of an arthrodesis cushion to limit deep hip flexion in clinical practice. However, the patients need to be educated on the correct use of the cushion and the hip brace, in order for them to work.

Ishii et al. reported that hip braces, in particular in the early phase after THA, when the most dislocations occur, help to remind patient not to position their hips in positions associated with increased dislocation risk [10]. Our study confirms the biomechanical effects of hip braces on limiting positions that might lead to THA dislocation. In contrast, DeWal et al. did not find a significant benefit of Hip braces on the re-dislocation rates of THA after reviewing 58 patients [3]. Our study is the first to add biomechanical data to the topic of hip brace use in patients. Further studies seem necessary to study the biomechanical and clinical effectiveness of hip brace in a large cohort of patients. Limitations of our study include the fact that we have measured young and healthy volunteers instead of patients after THA. This has to do with the fact that we did not want to provoke or risk a hip dislocation in THA patients. It is also possible that age-specific changes in the musculoskeletal system could lead to different results then when testing an older population. We believe that the basic biomechanical effects of a hip brace can also be tested in a healthy population. However, in future studies, we would like to use this testing setup to evaluate the effectiveness of hip braces in the setting of postoperative patients. Furthermore, it would also be of interest to test different types of hip braces and different settings during the here evaluated tasks. With the results of this study, we were able to demonstrate that inertial sensors can be used to evaluate the effectiveness of hip braces, so that in future studies it would be possible to measure even more complex tasks. Nonetheless, the inertial sensors limited the way we performed the control setting of our measurements. We used the unhinged brace setting instead of measurements without the hip brace.

This is due to the fact that to measure hip and pelvic motions in a standardized and repetitive fashion two sensors were placed over the hip brace itself (see Fig. 1) and that repositioning of the sensors would have led to sensor-based variabilities in our results. Therefore, in future studies it would be helpful to modify the testing setup so that we can also include reliable measurements without the hip brace on.

## Conclusion

Hip braces can significantly reduce hip ROM during different tasks as expected. However, the measured hip flexion angles were greater than the settings of the hip brace should have allowed. The results also show that the use of an additional cushion can further limit hip motion.
